# Novel Interventional Approaches for ALI/ARDS: Cell-Based Gene Therapy

**DOI:** 10.1155/2011/560194

**Published:** 2011-07-13

**Authors:** Ying-Gang Zhu, Jie-Ming Qu, Jing Zhang, Hong-Ni Jiang, Jin-Fu Xu

**Affiliations:** ^1^Department of Pulmonary Medicine, Huadong Hospital, Shanghai 200040, China; ^2^Shanghai Medical School of Fudan University, Shanghai 200032, China; ^3^Department of Pulmonary Medicine, Zhongshan Hospital, Shanghai 200032, China; ^4^Department of Pulmonary Medicine, Shanghai Pulmonology Hospital, Tongji University, Shanghai 200043, China

## Abstract

Acute lung injury (ALI) and its more severe form, acute respiratory distress syndrome (ARDS), continue to be a major cause of morbidity and mortality in critically ill patients. The present therapeutic strategies for ALI/ARDS including supportive care, pharmacological treatments, and ventilator support are still controversial. More scientists are focusing on therapies involving stem cells, which have self-renewing capabilities and differentiate into multiple cell lineages, and, genomics therapy which has the potential to upregulate expression of anti-inflammatory mediators. Recently, the combination of cell and gene therapy which has been demonstrated to provide additive benefit has opened up a new chapter in therapeutic strategy and provides a basis for the development of an innovative approach for the prevention and treatment of ALI/ARDS.

## 1. Introduction

Acute lung injury (ALI) and its more severe form, the acute respiratory distress syndrome (ARDS), are syndromes consisting of acute respiratory failure with bilateral pulmonary infiltrates due to intra- or extrapulmonary risk factors [1]. Despite advances in therapeutic principles, the incidence and outcome of ALI/ARDS are widely considered to have remained high. In a recent publication [2], the incidence of ALI and ARDS for patients 15 years of age or older in the United States, substantially underestimated in the past [3], was reported to be 78.9 and 58.7 per 100,000 persons per year, with a mortality rate of 38.5% and 41.1%, respectively. The incidence of ALI increased with age from 16 per 100,000 person-years for those 15 through 19 years of age to 306 per 100,000 person-years for those 75 through 84 years of age. Mortality increased with age from 24% for those 15 through 19 years of age to 60% for those 85 years of age or older. A prospective, multicenter study [4] performed in ICUs in Shanghai found a 2% incidence of ARDS for patients 15 years of age or older with a nearly 70% mortality rate. As reported data varied widely, a meta-analysis by Zambon and Vincent [5] published in 2008 concluded that the overall pooled mortality rate was 43% and there had indeed been a reduction in mortality rates, approximately 1.1%/year for patients with ALI/ARDS over the last decade. Although some anticipated trials supported a number of therapies (e.g., nitric oxide therapy [6], prone positioning [7], and extracorporeal membrane oxygenation (ECMO) [8, 9]) responsible for improvements in ALI/ARDS mortality, Moran et al. [10] argued that a null effect of ECMO was not excluded and there appeared only weak evidence of efficacy.

According to the American-European Consensus Conference (AECC) on ARDS [1, 11], the diagnostic criteria are the presence of acute hypoxemia with a ratio of the partial pressure of arterial oxygen to the fraction of inspired oxygen (PaO_2_ : FiO_2_) of 300 mmHg or less (for ALI) or of 200 mmHg or less (for ARDS); bilateral infiltrates on chest radiograph and no clinical evidence of left atrial hypertension or a pulmonary artery wedge pressure of 18 mmHg or less. It has been extensively adopted that the lung injury score or the Acute Physiology and Chronic Health Evaluation (Apache III) or Simplified Acute Physiology Score (SAPS II) scoring systems can be applied to assess the severity of ALI/ARDS and the associated clinical features [12, 13]. Nevertheless, the GOCA stratification system (gas exchange, organ failure, cause, and associated diseases) was recommended by AECC [11] to facilitate incorporation of additional clinical information and prediction of outcome.

Frequently, ALI/ARDS has been noticed to have systemic manifestations because its triggering conditions are systemic and impairment of the lung leads to sepsis or inflammatory response syndrome [14, 15]. Most studies [4, 5, 16, 17] have shown that patients with refractory ALI/ARDS died of multiple organ dysfunction syndromes (MODSs), rather than isolated respiratory failure. Till now there is no universal theory concerning the pathogenesis of ALI/ARDS, but it can be understood to be any cellular or molecular mechanism associated with inflammatory pathways implicated in lung injury. Some reviews [18, 19] supported the findings that the alveolar epithelium along with lung leukocytes and coagulation pathway play a key role in the formation and clearance of ALI/ARDS and outcomes can be improved by increasing the rate of liquid clearance through *β*-adrenergic stimulation. On the other hand, several compounds which are either synthesized by or act on endothelial cells have been used for lung injury treatment in experimental studies or in human trials.

The present therapeutic approaches for ALI/ARDS consist of supportive care, pharmacological treatments, and ventilatory support including high-frequency ventilation (HFV), inverse ratio ventilation (IRV), airway pressure release ventilation (APRV), positioning of the patients, exogenous surfactant administration, liquid mechanical ventilation and extracorporeal membrane oxygenation (ECMO), and carbon dioxide removal (ECCO_2_R). However, the benefit of innovative ventilatory strategies is still controversial [20]. Combined respiratory care and inhaled nitric oxide have not been shown rigorously to improve overall mortality [4]. A research [21] conducted by Slutsky and Tremblay revealed a vicious cycle that mechanical ventilation, although being an indispensable tool for providing adequate gas exchange and resting respiratory muscles in certain patients with ARDS, may contribute to the initiation and/or propagation of a systemic inflammatory response leading to MODS. At the heart of the dilemma, the question remains “what is the most effective way to recover multiple organ failure?” In an attempt to escape this vicious cycle, innovative approaches, cell and/or gene therapy, are now being contemplated. We searched several electronic databases including Medline through PubMed (1990 to 2011), EMBASE through OVID (1990 to 2011) and Web of Science (1990 to 2011) and also reviewed the Cochrane Library to find relevant articles and searched the reference lists from primary and review articles to find articles concerning cell and/or gene therapy for ALI/ARDS. This paper aimed to provide some background material and highlight the importance and promise of these new therapeutic strategies.

## 2. Cell Therapies for ALI/ARDS

Recently, therapy involving stem cells, which have clonogenic and self-renewing capabilities and differentiate into multiple cell lineages [22, 23] has become eye catching. The stem cells evaluated for this purpose include mesenchymal stem cells (MSCs) and embryonic stem (ES) cells. 

Studies have demonstrated that treatment with MSCs provides a significant survival advantage both by systemic administration in experimental models of bleomycin-induced lung injury [24, 25] and through intrapulmonary delivery in mice with endotoxin-mediated ALI [26, 27]. One explanation of persistent lung injury and fibrosis attenuation is that MSCs not only migrate to the termination of acute inflammation in the lungs and differentiate into organ-specific cells [28], but increase the production of growth factors that may mobilize endogenous stem cells. Both increased mobilization of endogenous progenitor cells and alterations in the local cytokine milieu in favor of repair may contribute to the protective effects of stem cell transplantation as outlined by Rojas et al. [25]. Another theory of protection holds that MSCs shift the balance from a proinflammatory to an anti-inflammatory response to endotoxin or bleomycin that includes enhanced production of IL-10 [26] and blocking TNF-*α* and IL-1 [29]. Despite this, there is strong evidence that progenitor cells do have the capacity to engraft and contribute to the mesenchymal compartment of the lung, the consequences of which may be either beneficial or harmful [30], depending upon the lineage of engrafting cells [31]. 

Embryonic stem cells have been regarded as another new perspective for cell therapy for ALI/ARDS. The capacity of ES cells to generate type II pneumocytes and cells of differentiated airway epithelial tissue, including basal cells, ciliated cells, intermediate cells, or Clara cells have been uncovered [32, 33]. Nevertheless, a major concern with the use of embryonic stem cells for therapeutic purposes is their potential to form teratomas in vivo; such tumorigenicity is thus far unknown for pluripotent adult progenitor cells [34].

In contrast to previous reports, Kotton et al. [35] provided the evidence of failure to detect reconstitution of lung alveolar epithelial cells by unfractionated bone marrow or purified hematopoietic stem cells after transplantation when autofluorescence and dead cells are excluded from analysis. This finding has been supported by Loi et al. [36] who demonstrated that bone marrow derived cells home to airway epithelium with a low frequency by current techniques. Further investigation, therefore, on the molecular mechanisms that govern marrow cell recruitment and phenotypic conversion is needed in order for this approach to be clinically feasible.

## 3. Gene Therapies for ALI/ARDS

More and more scientists are focusing on functional genomics approaches which have great potential to address questions related to changes in phenotypes of key cells in ALI/ARDS [37–39]. For example, injury mechanisms mediated by the release of granular enzymes or oxygen radicals by neutrophils, aggregation of neutrophils, monocytes and platelets, and activation of the clotting cascade use constitutive biochemical pathways and do not require induction of new transcripts or expression of new proteins. Furthermore, constitutively expressed but repressed messenger RNAs can be translated to proteins in response to cellular signals that are relevant to ALI/ARDS and its systemic manifestations. Thus, genomic approaches using microarrays and other methods have rapidly become major tools in biologic investigation and are increasingly being applied in ALI/ARDS. Among all the genomics strategies, one of the most effective one is to upregulate gene expression for anti-inflammatory mediators such as Endothelial Nitric Oxide Synthase (eNOS), inhibitory kappa B (I-*κ*B), Keratinocyte growth factor (KGF), and angiopoietin-1.

 Among the inflammatory reactions that lead to injury, nitric oxide (NO), synthesized by isoforms of NO synthase (NOS) such as endothelial NOS (eNOS), constitutive neuronal NOS (nNOS) and inducible NOS (iNOS), is an important factor that regulates microvascular permeability during the pathogenesis of ALI. It has been demonstrated [40, 41] that under chemical or inflammatory stress, increased NO production occurs primarily via the iNOS isoform. In addition, overabundance of NO causes autoinhibition of its own synthesis, with eNOS being more sensitive to feedback than the inducible isoform. Since the 1980s, extensive researches in the field of NO and ALI/ARDS have produced mixed results, adding to the uncertainty of manipulating the delicate balance between NO's therapeutic benefits and its toxic effects. Systematic reviews [42–44] failed to identify statistically significant beneficial effects of inhaled NO on clinical outcomes. Despite signs of transient improvement in oxygenation, inhaled NO may be hazardous since it may cause kidney function impairment. Therefore, recent studies have investigated the nonselective NOS and iNOS selective inhibitors. In contrast with the data supporting the inhibition of iNOS leading to the beneficial influence in animal models, nonselective inhibition of NOS has multiple adverse effects [41]. Thus, the potential roles of eNOS in ALI/ARDS need to be clarified. Although Yamashita et al. [45] previously reported that chronic eNOS overexpression results in resistance to lipopolysaccharide-(LPS-) induced endotoxin shock using eNOS transgenic mice overexpressing the bovine eNOS gene in endothelial cells, they modified the study of design by establishing a mechanical ventilation model using eNOS transgenic mice without stimulation of iNOS expression and activity and have still strengthened the notion that NO derived from chronic eNOS overexpression inhibited neutrophil-associated lung edema and inflammation by reducing cytokine production in lung injury [46]. Because of the distinction in response between the rodent and human model, the clinical effect and approach of eNOS delivery should be evaluated by further studies.

The transcription factor nuclear factor kappa B (NF*κ*B) is a central regulator of inflammatory and immune responses. It remains in an inactive form in the cytoplasm because of its association with inhibitory proteins (I*κ*Bs) and migrates to nucleus when NF*κ*B is activated by stimuli leading to phosphorylation and subsequent degradation of the I*κ*B proteins. Recently, it has been shown that overexpression of I*κ*B*α* or NF*κ*B decoy transfection decrease NF*κ*B activity and IL-8 secretion not only in endothelial and stromal cells and monocytes [47, 48], but in epithelial cells also [49]. Anti-inflammatory gene therapy based on nucleotide gene transfer may offer promise for ALI/ARDS but large clinical studies are yet lacking.

Keratinocyte growth factor (KGF) is an epithelial-specific growth factor secreted by fibroblasts and vascular smooth muscle cells. It has recently been discovered to be the principal mitogen for alveolar type II cells [50]. Baba et al. have demonstrated that transient overexpression of KGF in the lungs attenuate pathophysiological impairments in hyperoxia-induced acute lung injury in mice due to the proliferation of epithelial cuboidal cells and the increase of Ki67—and surfactant protein C (Sp-C)-positive cells [51]. This suggests that the protective properties of KGF are the product of multifactorial effects. In Stern et al.'s recent study [52], KGF was not detected in the BAL fluid of the 15 survivors except in one patient, whereas KGF was greater than the threshold in 13 of the 17 nonsurvivors patients. This is not contradictory with the results of previous studies [51, 53] in animals that demonstrated that intratracheal or intravenous administration of KGF protects against lung injury. However, in most experimental studies, KGF was given in large amounts (5 mg/kg), 48 or 72 hrs before the injury. It is, therefore, possible that the time of administration is a key factor. Elevation of KGF may occur too late in vivo, at a time where epithelial damage is already maximal. Actually, Stern et al. [54] have already reported that in ALI/ARDS human subjects, KGF is found at biologically active levels in the alveoli, acting in concert with parathyroid-hormone-related protein to regulate the apoptosis and cell proliferation balance in the alveolar epithelium. Thus, KGF gene transduction is supposed to be a potentially useful method to overcome the critical phase of ALI/ARDS despite the disadvantages of epithelial cell or type II cell hyperplasia related to the overexpression of KGF in the lungs [55].

As a ligand for the endothelial-specific receptor tyrosine kinase with immunoglobulin and epidermal growth factor homology domain 2 (Tie2), angiopoietin-1 (Ang1) plays a crucial role in embryonic vasculature development [56, 57]. It has been reported in experimental studies that Ang1 can act as an anti-inflammatory factor by reducing pulmonary inflammation, plasma extravasation, and the increase of cytokines and adhesion molecules. Some data have revealed that Ang1 ameliorates reactive-oxygen-species-(ROS-) induced ALI through attenuating vascular leakage and modulating the expression of inflammatory mediators [6, 58]. The clinical trial conducted by Van Der Heijden et al. [59] showed that Ang1 levels were lower in patients with ALI/ARDS than in controls. These findings provide a rationale that Ang1 can be a recommendable anti-inflammatory target for ALI/ARDS. However, the possible side effects of Ang1 treatment have been noticed lately, including the development of pulmonary hypertension, lymphatic sprouting, and proinflammatory and profibrotic effects in acute renal injury [60–62]. Therefore, future researches need to be focused on the two most studied Tie2 ligands Ang1 and Ang2—interferes with Ang-1 activation by preventing Ang-1 from binding to the receptor—to optimize therapeutic strategy for the treatment of ALI/ARDS.

## 4. Cell Based Gene Therapies for ALI/ARDS

Gene therapy is widely accepted to be a promising treatment option for both genetically determined and idiopathic diseases. However, the use of viral vectors has been problematic, ranging from host immune responses to tumorigenesis. To overcome the limitation of transient gene expression through classic adenoviral vectors, Grove et al. [63] used an approach of retrovirally transduced multipotent bone-marrow-derived stem cells (BMSCs) to deliver gene therapy to the lung epithelium and up to 20% of lung epithelial cells can be derived from BMSCs.

Therefore, a novel therapeutic approach, cell-based gene therapy with the combination of cell and gene therapies has proven to be successful in experimental pulmonary vascular disease from ALI/ARDS [64–66] or pulmonary hypertension [67, 68]. Histologically, human ALI/ARDS can be subdivided into the exudative phase and the subsequent fibroproliferative phase [69]. The exudative phase is characterized by a diffuse neutrophilic alveolar infiltrate with hemorrhage, the accumulation of macrophages and protein-rich pulmonary edema. Endothelial injury and subsequent loss of integrity of the endothelial barrier is a prerequisite for development of interstitial edema and increase in permeability. These insights into the mechanisms of injury and inflammation during ALI/ARDS have brought attention to therapeutic strategies targeting the pulmonary endothelium. In the work by McCarter et al. [64], they used skin fibroblasts cell-based gene therapy in a rat model of ALI to achieve targeted overexpression of Ang-1 in the lung microvasculature while circumventing the problems of in vivo transfection and avoiding the confounding effects of viral proteins. Overexpression of angiopoietin-1, a ligand of the endothelial-selective tyrosine-kinase receptor, along with protective enzymes of eNOS and heme oxygenase-1 (HO-1), sustained less lung injury. This therapeutic strategy could be advantageous over intravenous administration of recombinant Ang-1 protein, as it allows for more targeted expression of the transgene and overcomes issues of short protein half-life after injection. In their further investigation [65], there was an additional effect of Ang1 overexpressing MSCs compared with MSCs alone, not only on the extravasation of plasma proteins and inflammatory cells, but also on the levels of various inflammatory cytokines and chemokines in the BAL fluid. This synergistic effect of MSCs-based Ang1 gene transfer may be attributed to its ability to reduce endothelial cell activation and thus block the amplification of lung injury that is dependent on the recruitment of leukocytes from the pulmonary circulation into the injured lung. Nevertheless, it is clear that the benefit seen in response to cell-based therapy did not require a high level of long-term persistence of transplanted MSCs. This observation is consistent with a number of reports [70], which point to an important role for paracrine actions of transplanted cells on neovascularization and tissue healing. Interestingly, this benefit has also been supported in our research [66]. The LPS-induced lung injury was markedly alleviated in the group treated with MSCs carrying Ang1 compared with groups treated with MSCs or Ang1 alone. The expression of Ang1 protein in the recipient lungs was increased after MSCs-Ang1 administration. In addition, cells of MSC origin could be detected in the recipient lungs for 2 weeks after injection with MSCs. All the findings suggest that MSCs and Ang1 have a synergistic role in the treatment of LPS-induced lung injury, as MSC-based Ang1 administration may concentrate in the vessel-enriched lung tissue. However, the precise mechanism by which genetically engineered MSCs confer a therapeutic benefit in the model of ALI/ARDS remains to be determined. Besides, the endothelial progenitor cells (EPCs), a tissue engineer to reconstruct pulmonary vasculature, have been used to protect the tissue from ALI manipulated with gene transfer (such as nitric oxide, prostacyclin, and adrenomedullin) [15] since dysfunction of pulmonary vascular endothelium may play a role in the pathogenesis of pulmonary hypertension associated with ALI/ARDS. Zhao et al. [68] have reported that endothelial progenitor cells (EPCs) engineered to overexpress eNOS were significantly more effective than EPCs alone in a monocrotaline model of vascular injury, producing near complete reversal of established pulmonary hypertension. A number of clinical trials are required to assess the safety and efficacy of cell-based gene therapy in several different pathologies.

Despite the limitations of low percentage of the retained cells in lung and uncertainty of host immune response, the synergistic action of combined cell and gene therapy for ALI/ARDS not only allows direct targeting of the lung for clinical intervention, but also provides a site-specific source to release therapeutic proteins and/or other cellular products by the retained cells. Cell-based gene therapy for ALI/ARDS has opened up a new chapter in therapeutic strategy and provides a basis for the development of an innovative approach for the prevention and treatment of ALI/ARDS.

## 5. Conclusion

The acute lung injury (ALI) and its more severe form, acute respiratory distress syndrome (ARDS), continue to be a major cause of morbidity and mortality in critically ill patients. Interventional approaches such as cell therapy and gene therapy for ALI/ARDS have emerged while classic therapeutic strategies are still widely adopted. The combination of cell and gene therapy that has been demonstrated to provide additive benefits holds the promise of major contribution to the repair, replacement, and regeneration of damaged tissues and organs. Cell-based gene therapy, therefore, is supposed to be an expected approach to ALI/ARDS.
